# Acute Kidney Injury after Cardiac Surgery in Patients Without Chronic Kidney Disease

**DOI:** 10.21470/1678-9741-2018-0084

**Published:** 2018

**Authors:** Kátia Alves Ramos, Cristiane Bitencourt Dias

**Affiliations:** 1 Centro Universitário Patos de Minas (UNIPAM), Patos de Minas, MG, Brazil.; 2 Instituto de Assistência Médica do Servidor Público de São Paulo (IAMSPE), São Paulo, SP, Brazil.

**Keywords:** Acute Kidney Injury, Thoracic Surgery, Cardiac Surgical Procedures, Risk Factors, Mortality

## Abstract

**Introduction:**

Among patients undergoing cardiac surgery, the occurrence of acute renal injury appears to be associated with worse prognosis and increased mortality. The objective of this study was to evaluate risk factors and the impact this complication on mortality and survival after cardiac surgery among patients without chronic kidney disease.

**Methods:**

In this retrospective study, we reviewed the medical records of 142 patients who underwent elective coronary artery bypass grafting, valve replacement (single or multiple), or both (simultaneously) at a tertiary care hospital.

**Results:**

Among the 142 patients evaluated, the mean age was 58.28±13.87 years and 80 (56.33%) were female. The postoperative incidence of acute renal injury was 43.66%. Univariate analysis between the groups with and without acute renal injury revealed no significant differences, whereas multivariate analysis showed that risk factors for acute renal injury included valve replacement (OR=4.7, *P*=0.002, 95% CI=1.76-12.62, age (OR=1.044, *P*=0.012, 95% CI=1.01-1.07), previous cardiac surgery (OR=36.1, *P*=0.015, 95% CI=1.99-653.85), postoperative use of the vasoactive drug norepinephrine (OR=3.32, *P*=0.013, 95% CI=1.29-8.58) and dobutamine (OR=5.3, *P*=0.019, 95% CI=1.32-21.64). In our sample, there were 30 deaths, of which 25 had acute kidney injury. Survival was also lower among the patients with this complication, especially those who had required hemodialysis (OR=2.60, *P*<0.001, 95% CI=1.01-6.70) or had previously undergone cardiac surgery (OR=3.68, *P*<0.001, 95% CI=1.09-12.37).

**Conclusion:**

Our findings underscore the importance of identifying risk factors for developing acute renal injury after cardiac surgery, which can further the development of effective renoprotective strategies.

**Table:** 

Abbreviations, acronyms & symbols	
AKI	= Acute renal injury
AKIN	= Acute Kidney Injury Network
CABG	= Coronary artery bypass grafting
COPD	= Chronic obstructive pulmonary disease
CPB	= Cardiopulmonary bypass
ICU	= Intensive care unit
MAP	= Mean arterial pressure

## INTRODUCTION

Acute renal injury (AKI) is a severe complication that occurs in 3.5-31.0% of patients undergoing cardiac surgery, making it one of the most common complications observed in this group of patients^[1]^. In a study conducted by Kochi et al.^[2]^, the prevalence of postoperative AKI among cardiac surgery patients was 34.0%. In contrast, Santos et al.^[3]^, Chertow et al.^[4]^ and Conlon et al.^[5]^, reported rates of only 16.1%, 2.4%, and 1.1%, respectively. The considerable variation in the reported incidence and prevalence of AKI as a postoperative complication of cardiac surgery is due to differences in the diagnostic criteria adopted, as well as in study design, inclusion/exclusion criteria, patient profiles, and the type/number of treatment facilities involved^[6]^.

Evidence suggests that even slight postoperative increases in serum creatinine levels are associated with a significant increase in the risk of death^[7]^. Among individuals undergoing cardiac surgery, mortality has been reported to be as high as 8% and postoperative AKI can increase the mortality rate to over 60%^[7]^. The occurrence of AKI in patients undergoing cardiac surgery raises the mortality rate from 0.4-4.4% to 1.3-22.3%; when those same patients require dialysis, rates range from 25% to 88.9%, making severe postoperative AKI an independent risk factor for mortality that results in an 8-fold increase in the risk of death^[8,9]^. Therefore, cardiac surgery AKI is associated with serious complications as well as with prolonged intensive care unit (ICU) stays and with a worse quality of life. It also increases early and late mortality and health care expenditures^[10,11]^.

The early identification of patients at risk of developing AKI after cardiac surgery is an important strategy for improving the care of such patients during the intraoperative and postoperative periods. The following factors have been found to facilitate the development of AKI after cardiac surgery: age; obesity; female gender; valve replacement surgery; myocardial infarction in the last 30 days; low cardiac output; blood transfusion; prolonged cardiopulmonary bypass (CPB); the use of inotropic or vasoconstrictor drugs; the use of an intra-aortic balloon pump; diabetes mellitus; heart failure; chronic obstructive pulmonary disease (COPD); peripheral artery disease; systemic arterial hypertension; and chronic kidney disease^[3,8,12-15]^.

Epidemiological studies of AKI in cardiac surgery patients are important because they allow for better diagnosis of AKI and facilitate the prognosis estimation, as well as the development of new, more effective strategies to prevent and minimize this complication, thus reducing the associated morbidity and mortality^[16]^. Therefore, the objective of this study was to identify the risk factors for AKI in cardiac surgery patients without chronic kidney disease, as well as to assess the impact of AKI on the early mortality and survival of such patients, at a single cardiology center in the Southeastern region of Brazil.

## METHODS

### Study Population

This was a retrospective study of patients between 20 and 80 years of age who underwent coronary artery bypass grafting (CABG), valve replacement (single or multiple), or both (simultaneously), between January 2008 and January 2014. All the procedures had been performed at a tertiary care cardiology hospital that serves the public and private sector in the macro-region of Alto Paranaíba, in the Brazilian state of Minas Gerais.

### Exclusion Criteria

Patients who underwent cardiac surgery for congenital heart disease and emergency heart surgery were excluded. We also excluded patients who, within the last 72 hours before surgery, were injected with iodinated contrast (because of its potential nephrotoxicity), as well as those with a preoperative creatinine clearance < 60 mL/min (as calculated by the Cockcroft-Gault equation) and those who died within the first 24 hours after surgery. There was only one patient undergoing CABG surgery without CPB, so this case was excluded.

We reviewed the medical records of 195 patients who underwent cardiac surgery during the studied period. Of those 195 records, 51 were incomplete. Therefore, the final sample comprised 144 medical records. In the process of constructing the multivariate analysis (logistic regression) models, it was necessary to exclude two cases, because they were considered outliers in practically all analyses. Thus, the final analysis comprised 142 medical records. After analyzing the medical records, we contacted all individuals or the relatives by telephone, in order to obtain information about subsequent survival or mortality.

### Variables Studied

- For the preoperative period, we evaluated physical characteristics (gender, age, weight, and height), biochemical characteristics (prior complete blood count and baseline serum creatinine), and comorbidities (arterial hypertension, diabetes mellitus, coronary heart disease, and COPD), as well as the history of stroke, coronary artery disease, and cardiac surgery;
- For the perioperative period, we evaluated the type of surgery performed (CABG, valve replacement, or the combination of the two), the duration of CPB, the need for transfusion of blood products, and the use of vasoactive drugs (norepinephrine or dobutamine);
- For the postoperative period, we evaluated the results of the complete blood counts, serum creatinine, and serum urea—on postoperative days 1 and 2, as well as on the day of hospital discharge. We also evaluated mean arterial pressure (MAP), diuresis, and fluid balance on postoperative days 1 and 2, as recorded on forms filled out daily by the nursing staff;
- The need for blood transfusion, the use of vasoactive drugs (norepinephrine or dobutamine), the administration of diuretics, and the need for hemodialysis were evaluated;
- We noted the length of the ICU stay (in days) and whether the patient died or was discharged.

### Definition of Terms

- AKI was defined according to the Acute Kidney Injury Network (AKIN) classification, using serum creatinine and urine output as criteria for the evaluation of renal function. The AKIN classifies patients as stage 1, stage 2, or stage 3, according to the worst serum creatinine or urine output over a 48-hour period. Patients who require hemodialysis are classified as AKIN stage 3^[17]^;
- The preoperative period was defined as the time from the scheduling of the surgery to the arrival of the patient in the operating room;
- The perioperative period was defined as the time from the arrival of the patient in the operating room to ICU admission;
- The postoperative period was defined as the time from ICU admission to hospital discharge;
- Early mortality was defined as death occurring within the first 30 days after surgery;
- Late mortality was defined as death occurring ≥ 31 days after surgery up to the final follow-up period of three years;
- When necessary, hemodialysis was intermittent because this is the only type of service available;
- The research project was approved by the Research Ethics Committee (number 718380) of the Instituto de Assistência Médica ao Servidor Público (Institute for the Medical Care of Civil Servants) and registered at Plataforma Brasil (number CAAE 00555512.7.0000.5463).

### Statistical Analysis

All statistical analysis were performed on the IBM SPSS Statistics software package, version 22.0 (IBM Corporation, Armonk, NY, USA). The statistical analysis was carried out in two stages.

Continuous variables were compared with the Student's t-test, and categorical variables were compared with the chi-square test. Once these variables had been listed, multiple logistic regression was used in order to examine the influence of all variables simultaneously and their relation to the occurrence of AKI. In the process of constructing the multivariate analysis (logistic regression) models, it was necessary to exclude two cases, because they were considered outliers in practically all analyses. Therefore, the final analysis comprised 142 medical records.

In the second stage, we evaluated the impact of AKI on mortality and survival. We used the chi-square test to evaluate the impact of AKI on mortality and the Mantel-Haenszel method (common odds ratio) to evaluate the probability of death due to AKI in the postoperative period. Subsequently, the Kaplan-Meier method was applied to compare the patients who developed AKI after cardiac surgery with those who did not, in terms of survival, and Cox regression was used to determine if there were other variables that directly affected survival. The outcome measure (mortality) was evaluated for a maximum of 3 years after cardiac surgery. Values of *P*<0.05 were considered statistically significant.

## RESULTS

### Descriptive Analysis

In the sample (n=142), the mean age was 58.28±13.87 years, the mean body mass index was 26.05±5.82 kg/m^2^ and 80 (56.33%) were female. The most common surgical procedure was valve replacement alone, which was performed in 81 cases (57.05%), followed by isolated CABG, performed in 56 (39.43%), and the combination of the two, performed in 5 (3.52%). The mean duration of CPB was 54.58±20.60 minutes. The mean serum creatinine level was 1.09±0.29 mg/dL at baseline, 1.50±0.51 mg/dL on postoperative day 1, and 1.57±0.89 mg/dL on postoperative day 2. The mean serum urea level was 39.71±16.57 mg/dL at baseline, 51.80±21.72 mg/dL on postoperative day 1, and 66.48±27.56 mg/dL on postoperative day 2. Preoperative laboratory tests showed a mean hemoglobin of 12.98±6.55 g/dL and a mean hematocrit of 37.40±4.78%. On postoperative days 1 and 2, the mean hemoglobin was 10.92±7.42 g/dL and 9.66±1.72 g/dL, respectively, and the mean hematocrit was 31.24±5.05% and 29.31±5.17%, respectively.

Among the patients evaluated, the mean MAP on postoperative days 1 and 2 was 74.05±10.38 mmHg and 76.12±10.53 mmHg, respectively. On postoperative days 1 and 2, the mean urine output was 2476.13±1067.61 ml/day and 1437.25±968.92 ml/day respectively.

The frequency of AKI of any degree within the first 48 hours after cardiac surgery was 43.66%, AKI occurring in 62 patients, of whom 36 (58.06%) were classified as AKIN stage 1, whereas 5 (8.06%) were classified as AKIN stage 2, and 21 (33.8%) were classified as AKIN stage 3. Among the 62 patients who developed AKI, hemodialysis was required for 12 (19.35%). In the sample as a whole, the mean ICU stay was 4.4±2.4 days. The other descriptive variables are shown in Table 1.

**Table 1:** Descriptive analysis of the preoperative, perioperative, and postoperative variables studied in 142 patients undergoing cardiac surgery.

Variables	n (%)
Comorbidities	
Systemic arterial hypertension	67 (47.18)
Diabetes mellitus	39 (27.46)
History of cardiac surgery	10 (7.04)
History of coronary heart disease	61 (42.95)
History of stroke	3 (2.11)
Chronic obstructive pulmonary disease	18 (12.67)
Perioperative use of blood products and amines	
Fresh-frozen plasma	17 (11.97)
Packed red cells	3 (2.11)
Packed platelets	20 (14.08)
Cryoprecipitate	3 (2.11)
Norepinephrine	7 (4.92)
Dobutamine	6 (4.22)
Postoperative use of blood products and amines	
Packed red cells	41 (28.87)
Norepinephrine	48 (33.80)
Dobutamine	30 (21.12)
Diuretics	97 (68.30)

### Comparison Between the Patients With and Without AKI

The patients with AKI (n=62) did not differ significantly from those without AKI (n=80) in terms of the mean age—59.92±14.19 years *vs.* 57.01±13.57 years (*P*=0.18); the mean duration of CPB—50 (range, 40-70) minutes *vs.* 50 (range, 40-60) minutes (*P*=0.10); the mean preoperative serum creatinine—1.07 (range, 0.86-1.29) mg/dL *vs.* 1.00 (range, 0.90-1.24) mg/dL (*P*=0.99); and the mean preoperative serum urea—38.00 (range, 30.00-47.75) mg/dL *vs.* 35.50 (range, 29.25-43.00) mg/dL (*P*=0.36). We also found no significant difference between the patients with and without AKI in terms of the mean MAP on postoperative day 1—72.5 (range, 65.0-80.0) mmHg *vs.* 70.0 (range, 68.0-85.0) mmHg (*P*=0.36)—or on postoperative day 2—75.0 (range, 65.0-85.0) mmHg *vs.* 75.0 (range, 68.5-85.0) mmHg (*P*=0.36). On postoperative day 1, the patients with and without AKI did not differ significantly in terms of the mean fluid balance—100.0 (range, −301.0 to 880.8) mL *vs.* 288.0 (range, −250.0 to 1439.0) mL (*P*=0.34)—nor was there any significant difference between the two groups in terms of the perioperative transfusion of blood products, which occurred in 90.3% of the patients with AKI and in 82.5% of those without (*P*=0.22). In the postoperative period, norepinephrine was used in 30 (48.38%) of the 62 patients with AKI and in 18 (22.5%) of the 80 patients without (*P*=0.0008), whereas dobutamine was used in 23 (37.09%) of the 62 patients with AKI and in 7 (8.8%) of the 80 patients without (*P*<0.0001).

We found no significant difference between the patients with and without AKI in terms of the type of surgical procedure performed, isolated CABG being performed in 19 (30.64%) of the 62 patients with AKI and in 37 (46.25%) of the 80 patients without, valve replacement alone being performed in 40 (64.51%) and 41 (51.25%), respectively, and the combination of the two being performed in 3 (4.83%) and 2 (2.5%), respectively (*P*=0.11 for all) (Table 2). However, analyzing a possible relationship between the type of surgery and the need for the use of dobutamine in the postoperative period, we observed that a smaller number of patients undergoing CABG required dobutamine in the postoperative period than did those undergoing valve replacement or the combination of the two procedures (Table 3).

**Table 2:** Clinical and laboratorial differences between patients with and without acute kidney injury (AKI).

Clinical and laboratorial characteristics		Patients with AKI n=62	Patients without AKI n=80	*P*
Age (years)		59.92±14+19	57.01±13.57	0.18
Type of surgical procedure (%)	CABG	30.62	46.25	0.11
	Valve replacement	64.61	51.25	0.11
	Combination	4.83	2.56	0.11
CPB (minutes)		50 (40-70)	50 (40-60)	0.10
Preoperative serum creatinine (mg/dl)		1.07 (0.86-1.29)	1.00 (0.91-1.24)	0.99
Preoperative serum urea (mg/dl)		38 (30-47.75)	35.50 (29.25-43)	0.36
MAP (mmHg) day 1		72.50 (65-80)	70 (68-85)	0.36
MAP (mmHg) day 2		75 (65-85)	75 (68.50-85)	0.36
Fluid balance (ml)		100 (-301-880.80)	288 (-250-1439)	0.34
Perioperative transfusion of blood products (%)		90.30	82.50	0.22
Postoperative norepinephrine use (%)		48.38	22.50	0.0008
Postoperative dobutamine use (%)		37.09	8.80	<0.0001

CABG=coronary artery bypass grafting; CPB=cardiopulmonary bypass; MAP=mean arterial pressure

**Table 3:** Relationship between the type of cardiac surgery and the need for dobutamine use in the postoperative period.

Postoperative dobutamine use	CABG	Valve replacement	Both
(n=56)	(n=81)	(n=5)
Yes (n)	6	20	4
No (n)	50	61	1

CABG=coronary artery bypass grafting

*P*=0.0007 *vs.* valve replacement and the combination of both procedures.

There was no significant difference between the patients with and without AKI in terms of the proportion of individuals with diabetes mellitus, which was reported in 32.36% (n=20) and 23.75% (n=19), respectively (*P*=0.22). We also found no significant difference between the patients with and without AKI in terms of the number of patients with a history of heart surgery (n=7 *vs.* n=3, *P*=0.09).

### Multivariate Analysis (Logistic Regression)

We evaluated 142 medical records of patients who underwent cardiac surgery. The variables potentially associated with the risk of developing AKI in the postoperative period are shown in Table 4. We found that undergoing valve replacement was a risk factor for postoperative AKI, as were advanced age, having previously undergone cardiac surgery, and postoperative vasoactive drug use.

**Table 4:** Multivariate analysis of risk factors associated with the development of acute kidney injury after cardiac surgery.

Variable	β	S.E.	Wald	df	*P*	OR	95% CI
Age	0.043	0.017	6.257	1	0.012	1.044	1.01-1.079
CABG			10.303	2	0.006		1.00-1.000
Valve replacement	1.549	0.503	9.467	1	0.002	4.706	1.76-12.615
CABG + valve replacement	−0.427	1.262	0.115	1	0.735	0.652	0.05-7.741
History of cardiac surgery	3.586	1.478	5.892	1	0.015	36.106	1.99-653.851
Perioperative blood transfusion	−3.742	1.034	13.098	1	<0.001	0.024	0.00-0.180
Postoperative use of packed red cells	3.640	2.340	2.421	1	0.120	38.108	0.39-3738.351
Perioperative use of norepinephrine	3.376	1.764	3.662	1	0.056	29.264	0.92-928.379
Postoperative use of norepinephrine	1.201	0.484	6.156	1	0.013	3.322	1.29-8.582
Postoperative use of dobutamine	1.677	0.713	5.536	1	0.019	5.349	1.32-21.639
Constant	−4.152	1.191	12.148	1	0.000	0.016	0.00-0.162

CABG=coronary artery bypass grafting

### Impact on Mortality and Survival Analysis

During the three-year period evaluated, there were 30 deaths in our sample, 17 (56.66%) of those deaths occurring in the first 30 days after cardiac surgery. Early and late mortality were higher among the patients with AKI than among those without, early mortality occurring in 15 and 2 of the patients, respectively (*P*<0.04) and late mortality (death occurring ≥ 31 days after surgery) in 10 and 3 of the patients, respectively (*P*<0.04). The odds ratio estimation test showed that the risk of death was 2.5 times higher in the patients who developed AKI after cardiac surgery than in those who did not (OR=2.5, 95% CI=1.06-6.00, *P*<0.04).

**Fig. 1:**
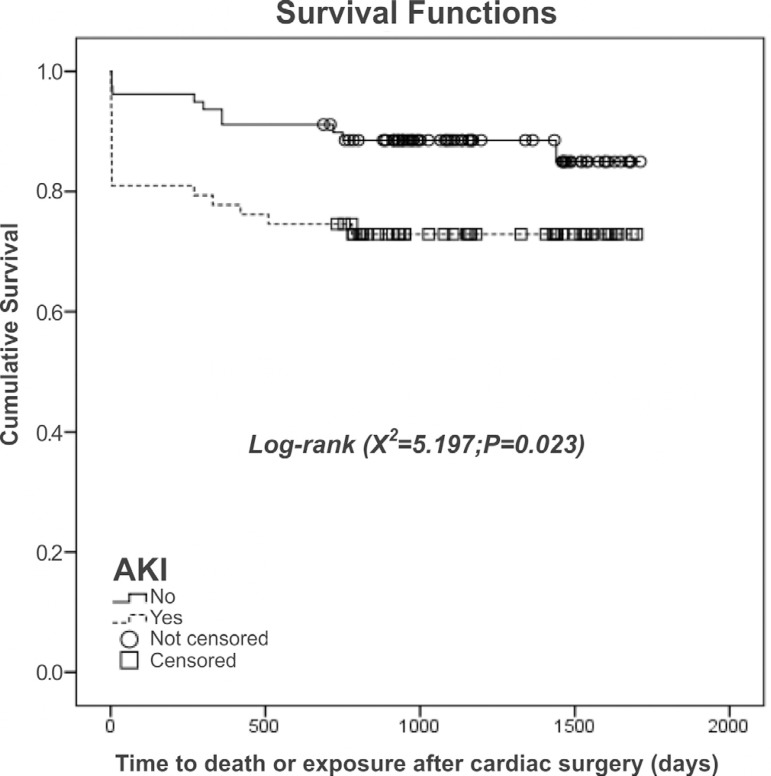
Survival analysis for individuals with and without acute kidney injury (AKI) after cardiac surgery.

**Fig. 2:**
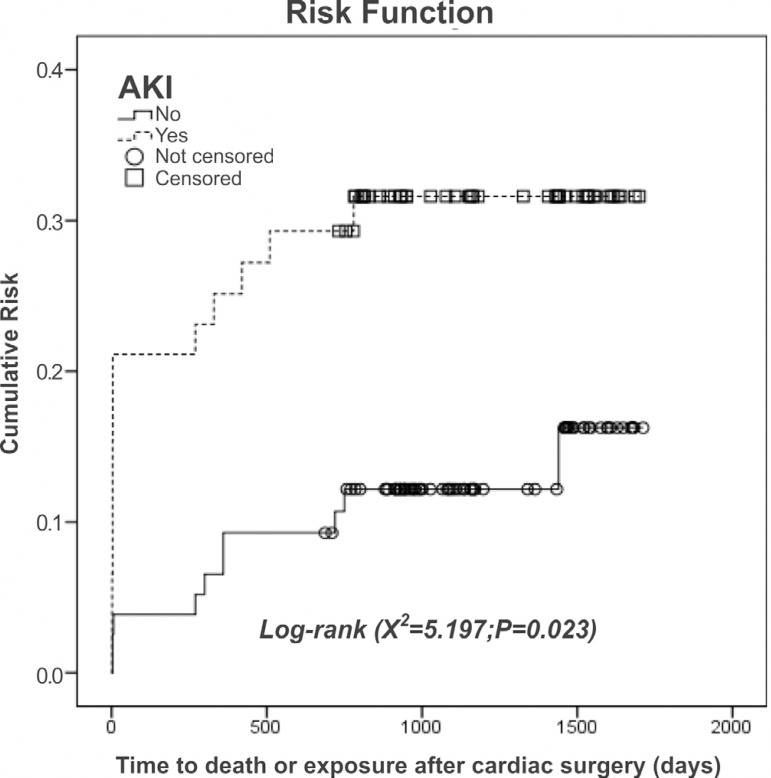
Analysis of early mortality among individuals with and without acute kidney injury (AKI) after cardiac surgery.

Consolidating the findings of our bivariate analysis, the Kaplan-Meier method demonstrated that in the first 30 days after surgery, individuals without AKI had a higher probability of survival, which persisted throughout the three-year follow-up period (Figure 1). The analysis of the risk of death (Figure 2) corroborated those findings, showing that individuals with AKI were more likely to die after cardiac surgery, either within the first 30 days after surgery or thereafter, than were those without AKI.

**Fig. 3:**
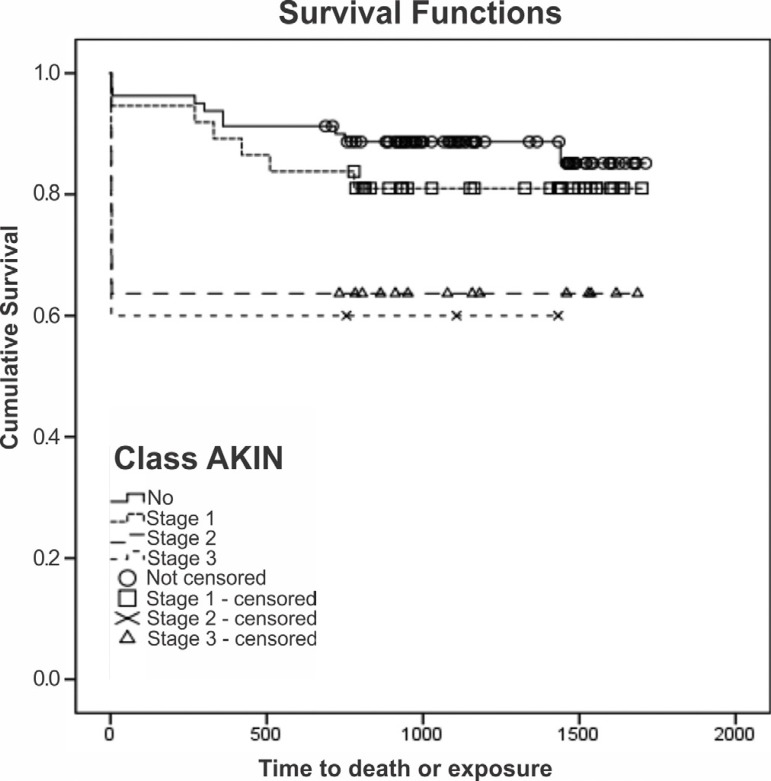
Analysis of survival according to the Acute Kidney Injury Network (AKIN) classification for acute kidney injury (AKI).

The Kaplan-Meier method showed that the survival rate was highest among the patients in which the AKI was classified as AKIN stage 1 and lowest among those in which it was classified as AKIN stage 3 (Figure 3). The Cox regression model showed that two variables had a negative impact on survival: having previously undergone cardiac surgery (OR=3.68, 95% CI=1.09-12.37, *P*<0.001); and requiring hemodialysis (OR=2.60, 95% CI=1.01-6.70, *P*<0.001).

## DISCUSSION

In the present study, we found that the incidence of AKI after cardiac surgery was 43.66%, much higher than the 16.4% and 15.7% reported by Andersson et al.^[14]^ and Kumada et al.^[18]^, respectively. That discrepancy could be attributed to the different definitions of AKI employed, variations in the methodologies, as well as in the patient profiles, and differences among the types of treatment facilities^[6,19]^. Our multivariate analysis showed that advanced age, valve replacement surgery, vasoactive drug use in the postoperative period, and having previously undergone cardiac surgery were the factors most strongly related to the development of AKI in a population from which patients with a creatinine clearance < 60 ml/min were excluded.

A progressive increase in the incidence of AKI among elderly patients has been reported in several studies^[3,20]^. According to Magro et al.^[20]^, that can be explained by the fact that the patients currently undergoing cardiac surgery are older and more critically ill than were those undergoing cardiac surgery in the past. Therefore, such patients have a worse prognosis and their recovery is delayed. Santos et al.^[3]^ found that being over 63 years of age is an independent risk factor for AKI. The loss of the renal functional reserve caused by the progressive reduction of the glomerular filtration rate with advancing age makes such patients more susceptible to renal damage when exposed to hypoperfusion^[6,21,22]^.

Our multivariate analysis demonstrated that patients undergoing surgery for valve replacement are at a greater risk of developing AKI than are those undergoing other types of cardiac surgery. Rodrigues et al.^[23]^ reported similar findings which can be explained by the fact that valve replacements are more complex procedures, with longer surgical times, which could have a direct effect on hemodynamic stability and on renal perfusion^[23]^. The idea of hemodynamic instability as the cause of AKI in valve replacement surgery was suggested in our study because dobutamine use in the postoperative period was found to be more common in valve replacements than in the other surgical procedures evaluated.

Our multivariate analysis showed that the patients who had previously undergone cardiac surgery were at a greater risk of developing AKI after a second cardiac surgery. In a study of 3,500 patients submitted to cardiac surgery, Karkouti et al.^[24]^ demonstrated that surgical re-exploration is associated with a number of negative outcomes, including AKI. However, the mechanisms involved have not been well elucidated.

In our sample, the postoperative use of amines (norepinephrine and dobutamine) was found to predispose to the development of AKI. Pontes et al.^[13]^, Lopez-Delgado et al.^[22]^, and Santos et al.^[3]^ also demonstrated that the use of inotropic drugs in the postoperative period was a determining factor for the development of AKI. This is likely associated with the mechanisms of renal ischemia and reperfusion caused by poor cardiac performance and hemodynamic instability^[3]^.

Although several studies have shown that prolonged CPB is a major risk factor for AKI^[14,22]^, that variable was found to be of little importance in our study, probably because the mean duration of CPB was quite short. Some studies^[12,24,25]^ have shown that blood transfusion during the perioperative period increases the likelihood that AKI will occur after cardiac surgery. However, in the present study, perioperative blood transfusion was shown to be mildly renoprotective, regardless of the type of blood products used. We can hypothesize that the perioperative transfusion of blood products improved the hemodynamic status of the patients in our sample and was therefore protective against AKI.

In the present study, AKI had a negative impact on patient survival, an effect that was more pronounced in the more advanced AKIN stages and in patients requiring hemodialysis. Various studies have shown that the development of AKI after cardiac surgery increases mortality, especially in patients who require dialysis^[13,22,23,26]^. In a study involving 3,240 patients undergoing cardiac surgery, Hobson et al.^[27]^ also analyzed survival rates in those who developed AKI in the postoperative period, reporting that one-year and five-year survival rates were lower for the patients who developed AKI (89% and 44%, respectively) than for those who did not (95% and 63%, respectively). Loef et al.^[28]^ corroborated those findings in a cohort of 843 patients who underwent cardiac surgery and were followed for 100 months thereafter. The authors found that, during the follow-up period, mortality was significantly higher in patients with postoperative deterioration of renal function and that survival remained lower regardless of whether there was recovery of renal function after hospital discharge. The mechanisms involved in this process are not fully understood, because AKI can be an early sign that other systems and organs are also negatively affected, and the sum of those dysfunctions could contribute to and explain the higher mortality and lower survival among individuals developing AKI after cardiac surgery.

However, our study has certain limitations: It is a retrospective study. In addition, the size of the patient sample was small, along with the fact that all the surgical procedures were performed at a single center, which could have limited the statistical power of the study.

## CONCLUSION

In the present study, we have shown that the occurrence of AKI after cardiac surgery had a negative influence on mortality and survival of the affected patients. Like other authors, we found that age, valve replacement, a history of cardiac surgery, and the use of vasoactive drugs in the postoperative period were independent predictors of the development of AKI after cardiac surgery in a population of patients with near-normal renal function. Thus, our findings underscore the importance of identifying risk factors for the development of AKI after cardiac surgery, which can further the development of effective renoprotective strategies.

**Table:** 

Authors' roles & responsibilities	
KAR	Substantial contributions to the conception or design of the work; or the acquisition, analysis, or interpretation of data for the work; final approval of the version to be published
CBD	Substantial contributions to the conception or design of the work; or the acquisition, analysis, or interpretation of data for the work; final approval of the version to be published
